# Effects of word length and word frequency among dyslexic, ADHD-I and typical readers

**DOI:** 10.16910/jemr.15.1.1

**Published:** 2022-06-14

**Authors:** Norberto Pereira, Maria Armanda Costa, Manuela Guerreiro

**Affiliations:** NeuroCog – Centro de Reabilitação da Lesão Cerebral; Center of Linguistics, School of Arts and Humanities, University of Lisbon; Escola Superior de Saúde Dr. Lopes Dias (ESALD); Instituto Medicina Molecular, Faculty of Medicine, University of Lisbon

**Keywords:** developmental dyslexia, attention-deficit/ hyperactivity disorder, eye tracking, eye movements, reading, length effects, lexical properties, group differences

## Abstract

This study aimed to investigate the neuropsycholinguistic functioning of children with Developmental Dyslexia (DD) and Attention-Deficit/Hyperactivity Disorder – inattentive subtype (ADHD-I) in a reading task. The psycholinguistic profile of both groups was assessed using a battery of neuropsychological and linguistic tests and compared to typical readers. Participants were submitted to a silent reading task with lexical manipulation of the text. Eye movements were recorded and compared aiming to find cognitive processes involved in reading that could help differentiate groups. The study examined whether word-frequency and word-length effects distinguish between groups. Participants included 19 typical readers, 21 children diagnosed with ADHD-I and 19 children with DD. All participants were attending 4^th^ grade and had a mean age of 9.08 years. Children with DD and ADHDI exhibited significant different cognitive and linguistic profiles on almost all measures evaluated when compared to typical readers. The effects of word length and word frequency interaction also differed significantly in the 3 experimental groups. The results support the multiple cognitive deficits theory. While the shared deficits support the evidence of a phonological disorder present in both conditions, the specific ones corroborate the hypothesis of an oculomotor dysfunction in DD and a visuo-spatial attention dysfunction in ADHD.

## Introduction

### Dyslexia

Developmental Dyslexia (DD) is a neurodevelopmental reading disability that adversely affects the speed and accuracy of word recognition, phonological processing, and as a consequence, impairs reading fluency and text comprehension, despite adequate instruction, and in the absence of general cognitive or sensory deficits (9; 10; 46; 58, 59). Dyslexics also exhibit distortions, substitutions, and omissions when reading aloud and silently (2). It is commonly estimated to affect between 5 to 10% of the population. Since reading ability is a skill that falls along a continuum, dyslexia is considered a difficulty along this continuum with no clear-cut or absolute limit. Thus, it is not possible to specify exactly how common dyslexia is, other than in relation to an approximate reader profile of what can be considered typical reading ability (9). Despite this uncertainty, there is good evidence for its neurobiological basis (76; 80), which reflects the fact that dyslexia occurs in varying degrees of severity (58; 81).

Although the causes of dyslexia are still not fully understood, and definitions and terminology vary, it is generally agreed that children who fail to acquire reading skill at a typical rate need careful monitoring and support during the early years of school (9).

Early identification and professional support is the most effective form of intervention for children with pronounced reading difficulties (58; 94). Linguistic parameters such as text complexity (67; 93), its syntax (95; 96), word length and word frequency (73; 92) can influence eye movements. Consequently, intervention strategies should account for the importance of perceptual parameters such as the properties of fonts (e.g., spacing), which are another aspect that has been shown to affect reading performance (20; 30; 33; 71; 84; 108).

To lessen the reading burden of dyslexic readers, designers have created dyslexia-friendly fonts such as OpenDyslexic69 and Dyslexie70. Nevertheless, these manipulations – whose fonts omit serifs, increase inter and intra-word spacing, and have unique letter strokes (26) – have not been found to increase reading speed (43; 47; 101).

Although most individuals are diagnosed during early school grades, the diagnosis can be made at any age (8). Fast, systematic and automated screening methods based on objective measurements of reading may help identify individuals at risk of dyslexia during the early school years (9; 57). Current methods, however, are limited in that they only measure individual cognitive skills that natural reading depends upon but say little about their interplay and function in actual reading (9). Invariably, these tests require the subject to produce some explicit response, typically under time pressure, such as marking the word boundaries in sequences of words without inter-word spaces, matching target words to corresponding pictures, or reading aloud pronounceable pseudowords of increasing difficulty. The proportion of correct responses gives an estimate of performance on a task related to reading but does not reflect the actual process of reading as it naturally occurs (9).

To overcome this limitation, we investigated the use of eye tracking during reading as a method for identifying and comparing different reading patterns among children with Dyslexia, ADHD–I and typical readers. By tracking eye movements during reading, it is possible to follow the reading process as it occurs in real-time and obtain objective measurements of this process. The data collected with this technique can provide a continuous record of reading that reflects both the speed and accuracy of the processes involved on text and word-based reading measures (36; 39; 60; 65; 68), in which reading accuracy can be determined from specific eye tracking data, such as regressions and go-past time.

Importantly, this type of measurement requires no overt response extraneous to the reading process itself and thus makes it possible to assess reading performance without placing additional task demands on the subject. As such, this approach differs in important ways from the screening methods currently in use.

Although it has long been known that the eye movements of children with dyslexia are different from those of typical readers, previous research has focused almost exclusively on identifying group-level differences (51; 53; 78).

### Attention-Deficit/Hyperactivity Disorder (ADHD)

ADHD is one of the most commonly diagnosed disorders in children with prevalence rates in the general population ranging from 3-7% (2). Assessing a child for ADHD can be difficult given the subjectivity of currently utilized assessment measures and the high degree of comorbidity between ADHD and other disorders (19). According to Willcutt et al. (105), ADHD and DD are two of the most common disorders of childhood, each occurring in approximately 5% of the population. Furthermore, ADHD and DD frequently co-occur, with a comorbidity typically ranging from 25 to 40% (23; 77; 90; 105, 103); boys with dyslexia have higher rates of comorbid externalizing disorders, including ADHD (104).

Students with ADHD often fall behind academically because of their attention problems. As a result of their poor academics, children with ADHD may appear to have a learning disability, such as a Reading Disability (RD). Additional deficits that children with ADHD may display in the school setting include poor rote memory, excessive vocalizations, difficulty delaying gratification, distractibility by extraneous stimuli, and difficulty listening and maintaining a conversation (6). These deficits, both individually and in combination, can make learning in the school setting very difficult (19).

The term ADHD-I will be used henceforth to refer only to that subgroup of this population whose topmost problem is inattention alone (predominantly inattentive type), which is the one that comprises our study. This subtype does not reflect a developmental deficiency in behavioural inhibition but probably one of focused/selective attention and speed of information processing (5; 7).

### Dyslexia Vs. ADHD

The vast majority of the research clearly demonstrates that dyslexia and ADHD–I represent two distinct clinical syndromes with separate cognitive profiles (106, 105, 102). Children with dyslexia exhibit deficits in phonological processing and other reading related skills while children with ADHD exhibit difficulties in executive functioning (56). Moreover, although not considered a primary deficit, difficulties in reading and listening comprehension have been associated with ADHD and likely contribute to their academic struggles (24; 25). Miller et al. (49) suggested that even when word reading ability was controlled, children with ADHD had difficulty building a coherent mental representation, and that difficulty was likely related to deficits in working memory.

These unique and distinctive deficits provide support for the validity of each diagnosis (19) and highlight the importance of assessing reading performance in children with ADHD.

### Eye Movements in Children with Dyslexia

Previous research has demonstrated that children with dyslexia exhibit different patterns of eye movements on reading tasks as compared to typical readers (19). While typical readers can read about 250 words per minute, the reading speed of children with dyslexia tends to be much slower because they make longer fixations, more frequent fixations, shorter saccades, and more regressions than typical readers (19). Longer fixations often occur because it takes them more time to comprehend information from the text. Children with dyslexia also have shorter saccades because they cannot cover as much information in their perceptual span (1; 66). Additionally, children with dyslexia tend to have unstable fixations and make more shorter saccades than typical readers (19). Dyslexics also process less parafoveal information on each fixation leading to more frequent and shorter saccades (65). Overall, these eye movement patterns are correlated with slower reading speed and poorer comprehension (28). Shorter saccades are common in letter-by-letter reading and contribute to a slow and laborious reading style, being the source of greater fixations among children with dyslexia as compared to typical readers (31). Hawelka and Wimmer (31) examined fixations, saccades, reading speed, and errors in reading in atypical and typical readers and found that the first group made fewer errors than normal readers, however, their reading speed was significantly slower than typical readers. These authors found that differences in reading rate were associated with the number of eye movements – fixations and saccades – made during reading. That is, participants with more eye movements had slower reading speeds (31).

### Eye Movements in Children with ADHD

Children with ADHD, analogous to children with dyslexia, may also have unique eye movement patterns, particularly regarding visual tracking tasks that require response inhibition of automatic saccadic eye movements (50). Munoz et al. (50), developed a prosaccade task in which ADHD and control participants ranging in age from 6 to 59 years old were asked to look at a target stimulus when it appeared on the screen and an antisaccade task where participants were asked to inhibit looking at the target stimulus. Results indicated that participants with ADHD displayed longer reaction times, more variability, and slower saccades in the prosaccade task compared to participants in the control group. In the antisaccade task, participants with ADHD had more difficulty inhibiting automatic saccades, displayed longer reaction times, and greater variability (50). In another study, children with ADHD – Combined subtype – and control children were compared to determine if eye movement data could be used to provide objective criteria for diagnosing ADHD (29). The eye movement task required children to remain focused on a fixation point that was stable for a period of 30 seconds and then moved back and forth on a computer screen. Results indicated that children with ADHD had greater difficulty maintaining fixations and made larger and more saccades than normal readers. There were no gender or age differences. It should also be noted that this task required visual tracking ability only and not reading skills specifically. Several studies which examined eye movements among children with ADHD, used eye movement paradigms that required tracking a visual stimulus rather than tasks that needed reading skills (40; 50).

In this study, we examined whether word-frequency and word-length effects would generalize equally to children with dyslexia, ADHD-I and typical readers. Word length and word frequency are two text characteristics which have a direct influence on eye movements of beginning readers during reading of connected text (11; 74, 72). Long length words usually receive longer and more fixations than short words (36; 39; 41) and infrequent words are fixated longer than frequent words (1Inhoff & Rayner, 1986; 67). Younger children show stronger length effects than older children (34), and dyslexic reading deficits in children also lead to stronger word length effects (35). Similarly, word frequency effects appear larger for children than for adults (12; 38). There is also some evidence for stronger word length effects for infrequent than frequent words in children’s eye movements (36; 61, 62) while the evidence for adults is less consistent (92).

However, according to Tiffin-Richards and Schroeder (92), only a few studies have used eye tracking methods with children in experimental designs to investigate the joint effects of word length and frequency on eye movements during reading. These authors sustain that the findings of those studies are mixed and may reflect differences in participant ages and reading ability as well as the nature of reading materials used in these studies. In addition, it is unclear whether findings can be generalized to children’s silent reading (3; 85; 87; 97) since, in a vast majority of studies done (34; 36; 61, 62), participants read aloud.

To investigate this question, we present empirical evidence from a silent reading experiment which focused specifically on the interaction of word length and word frequency effects in a sample of young readers. Hence, the present study sought to examine the eye movement patterns of children diagnosed with ADHD-I and DD during a reading task. It was hypothesised that: 1) children with dyslexia would exhibit longer fixation durations, more frequent fixations and higher number of regressive saccades than children with ADHD-I and normal readers, due to different neurocognitive and linguistic profiles; 2) children in the control group would exhibit a smaller number of regressive saccades, fewer fixations, and shorter duration fixations than children in the ADHD-I and DD groups; 3) children with DD and ADHD-I would be differently affected by the lexical properties of words presented in the text as compared to typical readers and, 4) there would be statistically significant differences in ocular movement patterns between DD and ADHD-I children as compared to typical readers.

## Methods

### Participants

The sample consisted of 59 Portuguese children, all were 9 years old (9.08±0.68), 61% female, native speakers of European Portuguese (L1) attending 4^th^ grade, distributed in three distinctive neuropsycholinguistic profiles, namely: 1) Control group (19 participants of whom 78.9% were female); 2) Children with dyslexia (19 participants of whom 57.9% were female) and 3) ADHD-I children (21 participants of whom 47.6% were female).

Control group inclusion criteria included: 1) Portuguese as first language; 2) a WISC-III full-scale IQ > 85; 3) absence of known neurological diseases; 4) absence of sensory (auditory or visual) or motor deficits; 5) exposure to adequate schooling; 6) medium-low minimum socioeconomical level, and 7) average or above average word reading skills assessed on a standardized test of reading fluency and accuracy. Dyslexia inclusion criteria included 1-6 criteria mentioned above plus a) experienced persistent problems in learning to read according to an independent assessment completed by the classroom teacher and, b) reading performance in the lower 5^th^ percentile of the full cohort on a standardized test of reading fluency and accuracy. ADHD-Inattentive subtype (ADHD-I) inclusion criteria included: 1) no comorbid pervasive developmental disorder, traumatic brain injury, or other neurological conditions; and 2) a WISC-III full-scale IQ > 75. ADHD-I children medicated with methylphenidate were excluded from the study. The diagnosis of ADHD-I and dyslexia was performed according to DSM-IV-R (2) diagnostic criteria. All children were administered the WISC-III as part of a neuropsychological evaluation (99, 100).

Neuropsychological and linguistic evaluations were carried out in Lisbon, Portugal. Eye movement recordings were collected at the Psycholinguistics Laboratory, School of Arts and Humanities, University of Lisbon.

Written informed consent was obtained from next of kin, caretakers, or guardians on behalf of the children enrolled in the study. The study protocol was approved by the Regional Ethical Review Board of the Faculty of Medicine in 2016, University of Lisbon.

Table 1 presents group means for age and IQ, while table 2 shows demographic characteristic according to gender.

**Table 1.** Group mean for age and IQ.

Measures	Control n*_i_* = 19		Dyslexia n*_i_* = 21		ADHD-I n*_i_* = 19	
	Mean	*SD*	Mean	*SD*	Mean	*SD*
Age	9.26	0.15	8.95	0.12	9.05	0.18
Verbal IQ	102.8	3.7	98.9	3.0	83.5	2.9
Performance IQ	103.0	4.5	98.2	2.4	80.9	1.9
Full IQ	102.5	4.0	97.5	2.5	78.5	1.9

Note. *SD* = Standard deviation.

**Table 2.** Demographic characteristic of the sample.

Sex					
Control group n*_i_*		Dyslexia n*_i_*		ADHD-I n*_i_*	
Male	Female	Male	Female	Male	Female
4	15	8	11	11	10

### Materials

Eye movements were recorded with SMI IVIEW X^TM^ HI-SPEED eye tracking system (SensoMotoric Instruments) (91). This video-based eye tracking compares the relative position of the pupil with the reflex coming from the cornea to calculate the ocular position at a sampling rate of 1250 Hz. This equipment was used to track eye position over time, sampling the horizontal and vertical position of the dominant eye (monocular). Under well controlled experimental conditions, the system afforded a tracking resolution of 0.01º with a gaze position accuracy of 0.25-0.5º, as per the manufacturer’s specification. Fixations were calibrated using 9-13 dots that randomly appeared in a 17-inch screen. The spatial accuracy of the equipment is 0.5º and to limit participant´s head movement a chin and forehead rest was deployed to minimize head movements and stabilize the viewing distance at 550 mm.

Word frequency in Portuguese language was determined using "Multifunctional Lexicon Computing of Contemporary Portuguese"(4) and ESCOLEX (86) databases. For frequency, words were divided in two intervals: 1) low-frequency words (LF) - [0-1000] Token and 2) medium-frequency words (MF) - [1001-10000] Token.

Regarding word-length, the criteria related to the size of the perceptual window and word size were the following (we have adjusted the criteria used by Hyönä & Olson, 36) to Portuguese: 1) short words (S) - [4-6] letters; 2) medium words (M) - [7-10] letters and 3) long words (L) - [11-14] letters (Table 3).

**Table 3.** Word classification according to their frequency and length.

	Stimuli	
Length	Short (S)	[4 - 6] Letters
	Medium (M)	[7 - 10] Letters
	Long (L)	[11 - 14] Letters
Frequency	Low (LF)	0 - 1000 Token
	Medium (MF)	1001 - 10000 Token
Length x Frequency	(S + LF)	Corais (corals)
	(S + MF)	Equipa (team)
	(M + LF)	Marinhas (marine)
	(M + MF)	Conhecer (to know)
	(L + LF)	Mergulhadores (sea divers)
	(L + MF)	Investigação (research)

### Procedure

We first determined the neuropsycholinguistic profile of each group. The neuropsychological and linguistic evaluations included instruments to assess intellectual performance (100), verbal working memory (digit span backward), short-term verbal memory (digit span forward), visual attention, phonological awareness (89), non-verbal fluid intelligence (13; 48; 63, 64; 82, 83), visuospatial ability and visuospatial memory (75), text comprehension (14) and, reading fluency and accuracy (17).

After this phase, each group was submitted to a reading task with control of text lexical properties and eye movements were recorded. Target words were distributed throughout the text to prevent them from being placed at the end of the paragraph and close to punctuation marks, which are positions favourable to wrap-up effects and, therefore, can be confused with words themselves. Also, contiguities between target words were avoided to mitigate spill over and agglomeration effects, that could hinder eye movement analysis. To improve readability and the posterior analysis of eye movement data, we selected Courier New, a non-proportional font, size 22; double line spacing was used in the final version of the text displayed on screen.

The reading task consisted of a text taken from the 2021 National Portuguese Language Final Examination, given to 4^th^ graders at the end of their school year. This text, entitled "120 new species discovered in Berlengas islands”, was subject to multiple changes at the level of its lexical, syntactic, and discursive properties. The objective was to reduce the level of complexity of the original text, so that it did not interfere with the lexical processing of the text. We gave preference to simple sentences and explicit correlational chains.

Our final goal was to devise a comprehensive eye movement account of reading profiles by investigating how eye movement patterns of children with dyslexia differ from ADHD-I children and, typical readers on global (text-based) and local (word-based) reading measures during an ecologically valid silent text reading task in Portuguese.

**Figure 1.**
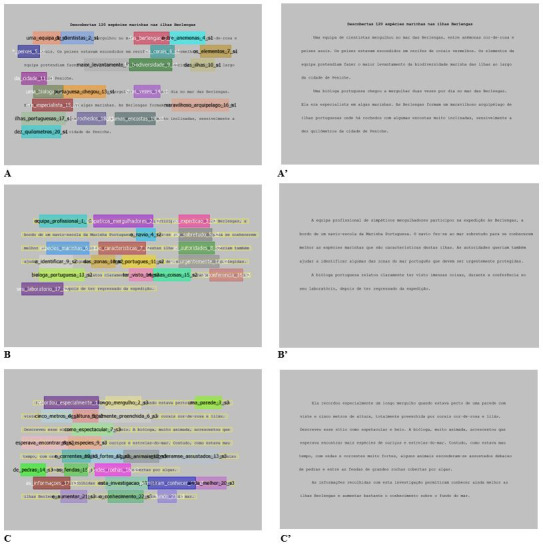
Reading task. A, B, and C are respectively 1^st^, 2^nd^ and 3^rd^ slides with coloured rectangles representing AOI / Target words. A’, B’ and C’ are respectively 1^st^, 2^nd^ and 3^rd^ slides with embedded target words. Total number of words = 264.

The text was divided in 3 pieces for presentation on a 17-inch screen (Figure 1: A’, B’ and C’). At the end of each slide, transition to the next slide was performed trough ocular fixation of the top right corner of the screen. The main experiment was preceded by a set of instructions and a pre-test. Monocular record of the dominant eye was recorded; ocular dominance was determined prior to the beginning of the experiment.

The pre-test training consisted of a silent reading task followed by three multiple-choice questions to determine the degree of text comprehension. The inclusion of a comprehension questionnaire at the end of each reading task served to ensure that participants identified the words, accessed their meaning, and integrated them into broader, syntactic, and discursive structures. The questions mainly served to encourage young readers to read for comprehension and to eliminate those who failed to answer 2 out of 3 questions. The comprehension outcomes were not used in any step of our analysis.

After this step, the equipment was once again calibrated according to the previously described specifications. The main reading experiment started afterwards.

Eye tracking data was collected at 1250Hz and stored offline for posterior analysis. To examine each target word as an Area of Interest (AOI), the following dependent variables were selected: Fixation Count (FC: number of fixations of all selected trials); Single Fixation Duration (SFD: the fixation duration of the fixation on a word, for AOIs in which only one fixation has been made); First Pass Reading Time (FPRT: sum of fixation durations from the first entry into an AOI until the eye leaves it in any direction), Second Pass Reading Time (SPRT: sum of fixation durations from the second entry into an AOI until the eye leaves it in any direction) and Total Fixation Time (TFT). The latter measure corresponds to the sum of FPRT and SPRT. AOIs for each target word were selected as represented in figure 1 (A, B and C).

In data analysis, to answer the hypotheses formulated, Frequency2 x Length3 interaction effects on eye tracking variables were measured through duration and frequency of fixations that landed on the target words, as also with FPRT and SPRT.

## Results

Participants eye movement behavior was determined using parametric and non-parametric statistics, by confirming normality assumption through *Shapiro-Wilk* normality test. Multivariate analysis was performed with *Anova F* statistic for equality of variances. In case of equality of variances, multiple comparisons were performed with *Tukey HSD* test. In the absence of equality of variances, *Brown-Forsythe* statistic was used as an alternative to the *Anova F* statistic using the *post-hoc Games-Howell* test. In case of normality violation assumption, *Kruskal-Wallis* test was used for independent samples. The assumptions for using the different statistical methods described above were as described in Marôco (2014) and Pestana & Gageiro (2014). Statistical analysis was performed using the IBM SPSS Statistics Version 25.

The results are presented in the following order: 1) Verbal Short-Term Memory; 2) Fixation Count (FC); 3) Single Fixation Duration (SFD); 4) Skipped Words; 5) First Pass Reading Time (FPRT); 6) Second Pass Reading Time (SPRT) and 7) Total Fixation Time (TFT).

No statistically significant differences between genders were found.

### Neuropsychological Profile 

WISC-III digit span subtest was subdivided in two additional variables to improve the search for group differences, namely digit span forward and digit span backwards; digit span subtest is included in WISC-III Verbal Working Memory Index (VWMI). Tables 4 and 11 (see Appendix) show that typical readers differed from children with dyslexia in digit span (F (2, 49) = 8.235, *p* = 0.001) and in digit span forward (F (2, 49) = 5.195, *p* = 0.009), both measures are linked to phonetic recoding.

Regarding the overall result achieved in digit span, we found statistically significant differences among children with dyslexia (*M* = 8.42, *SD* = 2.06; *n* = 19) and typical readers (*M* = 10.62, *SD* = 2.43; *n* = 13) (*p* = 0.023), the first group achieving worse results. Significant differences were also identified between typical readers and children with ADHD-I (*M* = 7.40, *SD* = 2.26; *n* = 20) (*p* = 0.027), the last group displaying an identical pattern as atypical readers. As far as digit span forward, statistically significant differences were found among children with dyslexia (*M* = 6.47, *SD* = 1.31; *n* = 19) and typical readers (*M* = 7.62, *SD* = 1.12; *n* = 13) (*p* = 0.041), and between typical readers and children with ADHD-I (*M* = 6.20, *SD* = 1.32; *n* = 20) (*p* = 0.008). Higher scores were obtained by typical readers. Lastly, digit span backwards allowed to find statistically significant differences within group distributions of this variable ( $X_{\text{KW}}^{2}\;$(2) = 6.237; N = 52; *p* = 0.044). Multiple comparison analysis showed that significant differences exist among typical readers and ADHD-I children (*p* = 0.042), with the first group achieving better results (see Table 4).

These results confirm that children with dyslexia have deficits in short-term verbal memory (articulatory loop), specifically in verbal working memory and attention, while children with ADHD-I additionally exhibited cognitive control and executive function deficits.

As for the remaining WISC-III subtests and composite results, it was observed that compared to typical readers, children with ADHD-I showed significant weaker cognitive performances in picture completion (*p* = 0.012), block design (*p* = 0.000), coding (*p* = 0.019), symbol search (*p* = 0.003), information (*p* = 0.036), vocabulary (*p* = 0.000), picture arrangement (*p* = 0.041), verbal scale IQ (*p* = 0.000), performance scale IQ (*p* = 0.000), full scale IQ (*p* = 0.000), Verbal Comprehension Index (VCI) (*p* = 0.000) and Perceptual Organization Index (POI) (*p* = 0.001) (see Appendix Tables 10 and 11).

Finally, the items that uniquely distinguished between children with dyslexia and children with ADHD-I were WISC-III similarities (*p* = 0.036), symbol-search (*p* = 0.003), vocabulary (*p* = 0.000), block design (*p* = 0.000), picture arrangement (*p* = 0.006), and coding (*p* = 0.015) subtests. Children with ADHD–I had overall worst results in all mentioned variables and composite results (see Appendix Tables 10 and 11).

As for Raven's Coloured Progressive Matrices (CPM-P), the mean total score achieved by children on this test differed significantly between children (F = (2, 49.778) = 4.631, *p* = 0.014), with differences among children with ADHD-I and typical readers (*p* = 0.009). There were also statistically significant differences between the distribution of set B across groups ( $X_{\text{KW }\;}^{2}$(2) = 10.238; N = 57; *p* = 0.006) especially between normative readers and children with ADHD-I (*p* = 0.005). In both cases, lower performances were observed in children with ADHD-I, which suggests that this group had several difficulties, namely in visuospatial reasoning, non-verbal abstraction capacity, visual attention, and language processing (see Appendix Table 12).

Finally, no significant differences were found between groups in Rey Complex Figure Test (RCFT), which means that visual-perceptual function did not interfere with reading skills (see Appendix Table 13).

In summary, the data suggests that there were more cognitive similarities between normative readers and children with dyslexia than between typical readers and children with ADHD-I.

### Linguistic Profile 

Data from the reading fluency and accuracy test, which assesses decoding, identification, integration, and production skills, made the detection of deficits common to dyslexia more evident. The results show that this test was highly discriminative, enabling to distinguish typical readers from children with dyslexia (*p* ≤ 0.001) and ADHD-I (*p* ≤ 0.001). No statistically significant differences were found between atypical readers and children with ADHD-I (see Appendix Table 14).

At last, reading comprehension test shows that children with ADHD-I, regardless of the type of comprehension assessed, were globally distinguished from typical readers by a worse performance in reading comprehension (F (2, 43) = 4.044, *p* = 0.025). This finding suggests that children with ADHD-I were more likely to make errors and respond impulsively given the nature of their attentional deficits. This overall effect at task level was not found between children with dyslexia and typical readers. Literal comprehension was the only level of comprehension that enabled to statistically distinguish between typical readers and children with dyslexia (F (2, 42) = 3.760, *p* = 0.031). This result suggests that children with dyslexia have low level of decoding and information integration because of difficulties in recognising the word, its phonological form and access to its meaning (see Appendix Table 15).

**Table 4.** WISC-III digit span subtest results.

Subtests	Groups	Mean (*SD*)	$\tilde{X}$(*Q3-Q1*)	Multiple comparisons
Digit Span Total	Control	10.62 (2.43)		Control group ≠ Dyslexia (*p* = 0.023)^1,2^ , Control group ≠ ADHD-I (*p* = 0.027)^1,2^
	Dyslexia	8.42 (2.06)		
	ADHD-I	7.40 (2.26)		
Digit Span Forward	Control	7.62 (1.12)		Control group ≠ Dyslexia (*p* = 0.041)^1,2^ , Control group ≠ ADHD-I (*p* = 0.008)^1,2^
	Dyslexia	6.47 (1.31)		
	ADHD-I	6.20 (1.32)		
Digit Span Backwards	Control		5.00 (5.00 – 4,00)	ADHD-I ≠ Control group (*p* = 0.042)^3^
	Dyslexia		3.00 (5.00 – 3,00)	
	ADHD-I		3.00 (4.00 – 3.00)	

Note. ^1^*ANOVA F statistic*; ^2^*Tukey HSD*;^3^*Kruskal-Wallis* independent samples test. *Q3* = 3^rd^ percentile. *Q1*= 1^st^ percentile. *SD* = Standard deviation. $\tilde{X}$ = Median.

### Fixation Count (FC)

**Table 5.** Median, 1^st^ and 3^rd^ percentiles, mean, standard deviation, and multiple comparison test: First Fixation Count as a Function of Word Frequency and Word Length interaction in Dyslexia, Control and ADHD-I Groups.

IV	Groups	$\tilde{X}$ *(Q3–Q1)*	Mean (*SD)*	*K-S*
S + LF	Control	2.00 (3.00 – 1.00)	2.12 (1.18)	Control group ≠ Dyslexia^*^
	Dyslexia	2.50 (4.00 – 2.00)	3.10 (1.97)	
	ADHD-I	2.00 (3.00 – 2.00)	2.44 (1.43)	
S + MF	Control	2.00 (2.00 – 1.00)	1.77 (0.90)	Control group ≠ ADHD-I^*^ , Control group ≠ Dyslexia^*^
	Dyslexia	2.00 (3.00 – 1.00)	2.69 (1.93)	
	ADHD-I	2.00 (3.00 – 1.00)	2.39 (1.58)	
M + LF	Control	3.00 (4.00 – 2.00)	3.10 (1.83)	Control group ≠ Dyslexia^*^ , ADHD-I ≠ Dyslexia^*^
	Dyslexia	4.00 (6.00 – 2.00)	4.74 (3.19)	
	ADHD-I	3.00 (4.00 – 2.00)	3.71 (2.51)	
M + MF	Control	2.50 (3.75 – 2.00)	2.75 (1.63)	Control group ≠ Dyslexia^*^ , ADHD-I ≠ Dyslexia^*^
	Dyslexia	4.00 (6.00 – 2.00)	4.45 (3.49)	
	ADHD-I	3.00 (4.00 – 2.00)	3.33 (2.16)	
L + LF	Control	3.50 (5.00 – 2.00)	4.05 (2.61)	Control group ≠ Dyslexia^*^ , ADHD-I ≠ Dyslexia^*^
	Dyslexia	6.00 (8.00 – 3.00)	5.64 (3.61)	
	ADHD-I	4.00 (6.00 – 2.00)	4.61 (3.21)	
L + MF	Control	3.00 (5.00 – 2.00)	3.57 (1.92)	Control group ≠ Dyslexia^*^
	Dyslexia	4.50 (7.00 – 3.00)	5.30 (3.83)	
	ADHD-I	3.00 (5.00 – 2.00)	4.52 (3.01)	

Note. S = Short word. M = Medium word. L = Long word. LF = Low frequency. MF = Medium frequency. *K-W* = *Kruskal-Wallis* test. $\tilde{X}$ = Median. *Q3* = 3^rd^ percentile. *Q1*= 1^st^ percentile. *SD* = Standard deviation. Superscripts indicate significant group difference; *p < 0.05

The eye tracking independent variables that showed statistically significant differences between one or more groups were: 1) Low-Frequency Short words (S + LF); 2) Medium-Frequency Short words (S + MF); 3) Low-Frequency Medium words (M + LF); 4) Medium-Frequency Medium words (M + MF); 5) Low-Frequency Long words (L + LF) and 6) Medium-Frequency Long words (L + MF).

#### Low-Frequency Short words (S + LF)

We found statistically significant differences between the distribution of the variable S + LF across groups ( $X_{\text{KW}}^{2}\;$(2) = 1.934; N = 409; *p* = 0.000). Multiple comparison analysis shows significant differences between typical readers and children with dyslexia (*p* = 0.000). The latter displaying the highest number of fixations counts in low-frequency short words (Table 5). This data suggests that typical readers were not significantly different from ADHD-I children.

#### Medium-Frequency Short words (S + MF)

We found statistically significant differences between the distribution of the variable S + MF across groups ( $X_{\text{KW}}^{2}$ (2) = 20.443; N = 420; *p* = 0.000). Multiple comparison analysis shows significant differences between typical readers and children with dyslexia (*p* = 0.000) and, between typical readers and ADHD-I children (*p* = 0.005). In both cases, typical readers had the lowest number of fixations counts in medium-frequency short words (Table 5).

#### Low-Frequency Medium words (M + LF)

Statistically significant differences were found between the distribution of the variable M + LF across groups ( $X_{\text{KW}}^{2}$ (2) = 23.275; N = 430; *p* = 0.000). Multiple comparison analysis shows significant differences between typical readers and children with dyslexia (*p* = 0.000) and, between children with dyslexia and children with ADHD-I (*p* = 0.012). Dyslexics had the highest number of fixations counts in low-frequency medium words (Table 5).

#### Medium-Frequency Medium words (M + MF)

We found statistically significant differences between the distribution of the variable M + MF across groups ( $X_{\text{KW}}^{2}$ (2) = 23.943; N = 430; *p* = 0.000). Multiple comparison analysis shows significant differences between typical readers and children with dyslexia (*p* = 0.000) and, between children with ADHD-I and children with dyslexia (*p* = 0.025). The latter group had the highest number of fixations counts in medium-frequency medium length words (Table 5).

#### Low-Frequency Long words (L + LF)

**Figure 2.**
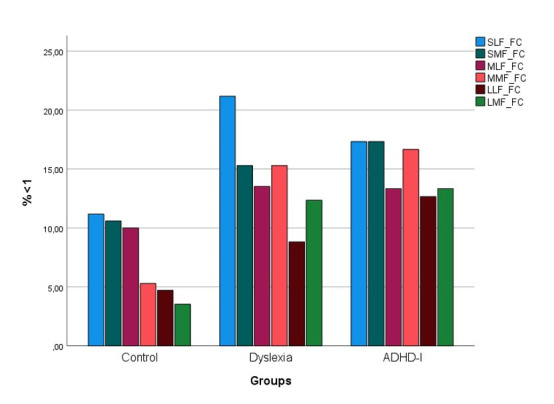
Percentage of skipped target words. Note. S = Short words. M = Medium words. L = Long words. LF = Low frequency. MF = Medium frequency.

Statistically significant differences were found between the distribution of the variable L + LF across groups ( $X_{\text{KW}}^{2}$(2) = 18.768; N = 448; *p* = 0.000). Multiple comparison analysis reveals significant differences between typical readers and children with dyslexia (*p* = 0,000) and, between children with ADHD-I and children with dyslexia (*p* = 0.024) (Table 5). In both cases, children with dyslexia had the highest number of fixations counts in low-frequency long words and, simultaneously, higher number of skipped words (Figure 2). This finding suggests that typical readers do not significantly differ from children with ADHD-I.

#### Medium-Frequency Long words (L + MF)

We found statistically significant differences between the distribution of the variable L + MF across groups ( $X_{\text{KW }\;}^{2}$(2) = 14.682; N = 443; *p* = 0.001). Multiple comparison analysis shows significant differences between typical readers and children with dyslexia (*p* = 0.000) (Table 5). Once again, children with dyslexia had the highest number of fixations counts in medium-frequency long words. This finding also suggests that typical readers were not significantly different form ADHD-I children.

Finally, figure 2 allows to conclude that children with ADHD-I had the highest percentage of skipped words as far as this measure is concerned, making it a strong discriminant variable.

Synthesizing, FC has a strong discriminative power to statistically distinguish between typical and dyslexic readers since there were significant differences between groups in all six-word conditions, with typical readers making fewer fixations than atypical readers. It also distinguishes between dyslexia and ADHD-I in two conditions of low frequency (medium and long length words) and one of medium frequency (medium length word).

### Single Fixation Duration (SFD)

**Table 6.** Median and Mean SFD duration (in milliseconds) on short x medium frequency target words in Dyslexia, Control and ADHD-I Groups.

Groups	Medium frequency (MF)		*K-W*
	Short (S)		
	$\tilde{X}$ *(Q3–Q1)*	Mean (*SD*)	
Control	290.00 (343.75-234.75)	301.04 (117.83)	Control group ≠ Dyslexia^*^
Dyslexia	375.50 (499.75-266.75)	393.50 (189.14)	
ADHD-I	304.00 (386.75-229.00)	329.05 (148.85)	

Note. *K-W* = *Kruskal-Wallis* test. $\tilde{X}$ = Median. *Q3* = 3^rd^ percentile. *Q1* = 1^st^ percentile. *SD* = Standard deviation. Superscripts indicate significant group difference (*p* < 0.05); * *p* < 0.00

The eye-tracking measure that produced the most significant group difference for SFD was S + MF (Medium-frequency Short Words). According to Kruskal-Wallis, statistically significant differences were found between group distributions of the variable S + MF ( $X_{\text{KW}}^{2}$ (2) = 8.329; N = 158; *p* = 0.016). Multiple comparison analysis shows that significant differences exist between typical readers and children with dyslexia (*p* = 0.012). The latter group had more single fixation durations in medium-frequency short words (Table 6). This finding suggests that children with dyslexia had higher lexical activation times in medium-frequency short words, a phenomenon that occurs at the early stages of word processing.

### Skipped words

Regarding the number of skipped words (see Figure 2), we can observe that children with dyslexia ignored the highest percentage of low-frequency short words. Regarding low-frequency medium words, this variable recruits more attention mechanisms to aid grapheme-phoneme decoding in children with dyslexia. This phenomenon was also observed in low-frequency long words, whose characteristics attract visual attention resources in children with dyslexia due to their uncommon lexical properties.

Furthermore, as word length increases, the number of target words skipped by typical readers decreases; the effect of word-frequency was emphasized in medium size words. Compared to typical readers, children with dyslexia and ADHD-I had globally higher numbers of skipped words. The latter group had the lowest sensitivity to linguistic variables, while children with dyslexia were more sensible to the combined properties of size and frequency interaction effects. Finally, figure 2 allows to conclude that medium-frequency short words were the most skipped by children with ADHD-I.

In summary, figure 2 shows that: 1) compared to typical and dyslexic readers, children with ADHD-I had consistently more skipped words in all conditions, which was expected given their visual attention deficits; 2) short and low-frequency words were the most skipped by children with dyslexia and, finally, 3) typical readers, as expected, exhibited a constant relationship between fixated words and their length. The shorter and familiar the words were, the more they were skipped, probably because typical readers have the capacity to perceive and recognize them in parafoveal vision.

### First Pass Reading Time

**Table 7.** Median, 1^st^ and 3^rd^ percentiles, mean, standard deviation, and multiple comparison test: First Pass Reading Time (in milliseconds) as a Function of Word Frequency and Word Length interaction in Dyslexia, Control and ADHD-I Groups.

IV	Groups	$\tilde{X}$ *(Q3–Q1)*	Mean (*SD*)	*K-S*
S + MF	Control	302.00 (411.00 – 199.50)	332.88 (183.79)	Control group ≠ Dyslexia^*^
	Dyslexia	439.00 (615.75 – 214.25)	486.62 (403.43)	
	ADHD-I	315.00 (548.00 – 187-00)	416.67 (302.55)	
M + LF	Control	395.00 (655.50 – 285.00)	566.44 (567.97)	Control group ≠ Dyslexia^*^
	Dyslexia	570.50 (1222.25 – 261.75)	954.28 (1167.07)	
	ADHD-I	485.00 (1119.50 – 257.00)	784.19 (758.42)	
M + MF	Control	352.00 (572.00 – 219.00)	432.22 (448.01)	Control group ≠ Dyslexia^*^
	Dyslexia	484.00 (999.50 – 244.50)	726.99 (752.77)	
	ADHD-I	380.00 (732.50 – 229.50)	583.07 (566.53)	
L + MF	Control	350.00 (609.50 – 153.00)	502.15 (593.30)	Control group ≠ Dyslexia^*^, Control group ≠ ADHD-I*
	Dyslexia	423.00 (1097.75 – 157.00)	966.61 (1280.98)	
	ADHD-I	448.00 (1100.50 – 198.50)	806.50 (891.14)	

Note. S = Short word. M = Medium word. L = Long word. LF = Low frequency. MF = Medium frequency. *K-W* = *Kruskal-Wallis* test. $\tilde{X}$ = Median. *Q3* = 3^rd^ percentile. *Q1* = 1^st^ percentile. *SD* = Standard deviation. Superscripts indicate significant group difference; *p < 0.05

The eye tracking independent variables that produced statistically significant differences between groups were: 1) medium-frequency short words (S + MF); 2) low-frequency medium words (M + LF); 3) medium-frequency medium words (M + MF) and, 4) medium-frequency long words (L + MF).

#### Medium-frequency short words (S + MF)

Kruskal-Wallis analysis shows statistically significant differences between the distribution of the variable S + MF across groups ( $X_{\text{KW}}^{2}$(2) = 13.104; N = 420; *p* = 0.001). Multiple comparisons analysis indicates that there were significant differences between normative children and children with dyslexia (*p* = 0.001), with the latter group having the highest FPRT observed in medium-frequency short words (Table 7).

#### Low-frequency medium words (M + LF)

We found statistically significant differences between the distribution of the variable M + LF across groups ( $X_{\text{KW}}^{2}$(2) = 8.666; N = 430; *p* = 0.013). Multiple comparisons analysis indicates that there were significant differences between normative children and children with dyslexia (*p* = 0.015). The latter group had higher FPRT for low-frequency medium words (See Table 7).

#### Medium-frequency medium words (M + MF)

Kruskal-Wallis points out significant differences between the distribution of the variable M + MF across groups ( $X_{\text{KW }\;}^{2}$(2) = 11.577; N = 430; *p* = 0.003). Multiple comparisons test indicates that there were statistical differences between typical readers and children with dyslexia (*p* = 0.002), with the latter group showing the highest FPRT for medium-frequency short words (Table 7).

#### Medium-frequency long words (L + MF)

We found statistically significant differences between the distribution of the variable L + MF across groups ( $X_{\text{KW}}^{2}$(2) = 9.833; N = 443; *p* = 0.007). Multiple comparisons analysis indicates that there were significant differences between typical readers and children with dyslexia (*p* = 0.033), and between typical readers and children with ADHD-I (*p* = 0.015). Moreover, children with ADHD-I had the highest first reading times for medium-frequency long words (Table 7).

In summary, the average duration of FPRT, considered a measure of slower linguistic processes when compared to lexical activation, was correlated both with word-frequency and comprehension processes that integrate several words, the latter related to the integration of grapho-phonological information and access to meaning. This finding suggests that children with dyslexia had longer reaction times, especially in low-frequency words.

### Second Pass Reading Time

**Table 8.** Median, 1^st^ and 3^rd^ percentiles, mean, standard deviation, and multiple comparison test: Second Pass Reading Time (in milliseconds) as a Function of Word Frequency and Word Length interaction in Dyslexia, Control and ADHD-I Groups.

ID	Groups	$\tilde{X}$ *(Q3–Q1)*	Mean (*SD*)	*K-S*
S + LF	Control	307.00 (505.00 – 167.00)	356.56 (210.75)	Control ≠ Dyslexia^*^, ADHD-I ≠ Dyslexia^*^
	Dyslexia	587.00 (1036.25 – 361.50)	864.32 (702.58)	
	ADHD-I	404.50 (623.75 – 239.50)	522.09 (453.17)	
S + MF	Control	305.50 (415.00 – 192.50)	334.45 (204.94)	Control ≠ ADHD-I^*^, Control ≠ Dyslexia^*^
	Dyslexia	482.00 (936.00 – 265.00)	656.95 (540.22)	
	ADHD-I	416.00 (764.00 – 235.00)	662.43 (693.62)	
M + LF	Control	543.50 (862.00 – 289.50)	629.51 (428.57)	Control ≠ Dyslexia^*^
	Dyslexia	820.50 (1774.75 – 378.25)	1264.86 (1228.22)	
	ADHD-I	885.00 (1599.00 – 284.00)	1080.73 (989.89)	
M + MF	Control	414.50 (696.50 – 298.00)	559.47 (445.82)	Control ≠ ADHD-I^*^, Control ≠ Dyslexia^*^
	Dyslexia	1001.00 (1563.00 – 338.00)	1297.95 (1480.99)	
	ADHD-I	749.00 (1188.50 – 337.00)	895.35 (728.23)	
L + LF	Control	575.00 (982.00 – 387.50)	816.82 (780.36)	Control ≠ ADHD-I^*^, Control ≠ Dyslexia^*^
	Dyslexia	1039.00 (2270.75 – 498.25)	1651.13 (1582.05)	
	ADHD-I	931.50 (1790.25 – 394.75)	1318.07 (1228.36)	
L + MF	Control	529.00 (860.50 – 289.00)	639.91 (463.04)	Control ≠ Dyslexia^*^, Control ≠ ADHD-I^*^
	Dyslexia	783.00 (1429.25 – 412.00)	1120.06 (1095.02)	
	ADHD-I	863.00 (1475.00 – 410.00)	1179.63 (1143.87)	

Note. S = Short word. M = Medium word. L = Long word. LF = Low frequency. MF = Medium frequency. *K-W* = *Kruskal-Wallis* test. $\tilde{X}$ = Median. *Q3* = 3^rd^ percentile. *Q1* = 1^st^ percentile. *SD* = Standard deviation. Superscripts indicate significant group difference; *p < 0.05

The eye tracking measures that exhibited statistically significant differences between one or more groups were: 1) low-frequency short words (S + LF); 2) medium-frequency short words (S + MF); 3) low-frequency medium words (M + LF); 4) medium-frequency medium words (M + MF); 5) low-frequency long words (L + LF) and 6) medium-frequency long words (L + MF). In a first observation, we would say that SPRT – as a late measure that captures lexical access processes and word integration into syntactic structures – had a strong discriminative value because it distinguished, in all condition’s, normal readers from children with dyslexia and, in the 3 conditions of word size medium frequency, ADHD-I from controls.

#### Low-frequency short words (S + LF)

We found statistically significant differences between the distribution of the variable S + LF across groups ( $X_{\text{KW}}^{2}$(2) = 28.815; N = 183; *p* = 0.000). Multiple comparisons test shows that there were significant differences between typical readers and children with dyslexia (*p* = 0.000) and, between children with ADHD-I and children with dyslexia (*p* = 0.007). In both cases, children with dyslexia had larger SPRT in low-frequency short words (Table 8).

#### Medium-frequency short words (S + MF)

We found statistically significant differences between the distribution of the variable S + MF across groups ( $X_{\text{KW}}^{2}$(2) = 11.278; N = 140; *p* = 0.004). Multiple comparisons analysis shows that there were significant differences between typical readers and children with dyslexia (*p* = 0.007) and, between normative readers and children with ADHD-I (*p* = 0.012). This measure shows that children with dyslexia produced higher SPRT in medium-frequency short words when compared to the remaining groups (Table 8).

#### Low-frequency medium words (M + LF)

We found statistically significant differences between the distribution of the variable M + LF across groups ( $X_{\text{KW}}^{2}\;$(2) = 10.106; N = 205; *p* = 0.006). Multiple comparisons analysis indicates that there were significant differences between normative children and children with dyslexia (*p* = 0.006). The latter group had higher SPRT for low-frequency medium size words (Table 8).

#### Medium-frequency medium size words (M + MF)

We found statistically significant differences between the distribution of the variable M + MF across groups ( $X_{\text{KW}}^{2}$(2) =16.616; N = 204; *p* = 0.000). Multiple comparisons test shows that there were significant differences between typical readers and children with dyslexia (*p* = 0.000) and, between typical readers and children with ADHD-I (*p* = 0.019). When compared to the remaining groups, children with dyslexia had higher SPRT in medium-frequency medium length words (See Table 8).

#### Low-frequency long length words (L + LF)

Statistically significant differences were found between the distribution of the variable L + LF across groups ( $X_{\text{KW}}^{2}\;$(2) = 14.021; N = 221; *p* = 0.001). Multiple comparisons analysis shows that there were significant differences between normative children and dyslexic readers (*p* = 0.001) and, between typical readers and children with ADHD-I (*p* = 0.032). Moreover, children with dyslexia exhibited higher SPRT in low-frequency long length words (See Table 8).

#### Medium-frequency long length words (L + MF)

We found statistically significant differences between the distribution of the variable L + MF across groups ( $X_{\text{KW}}^{2}$ (2) = 13.115; N = 239; *p* = 0.001). Multiple comparisons analysis shows that there were significant differences between typical readers and children with dyslexia (*p* = 0.007) and, also between typical readers and children with ADHD-I (*p* = 0.005). When compared with the remaining groups, children with dyslexia had higher SPRT in medium-frequency long length words (Table 8).

The results listed above clearly demonstrate that, compared to normal readers and children with ADHD-I, children with dyslexia had longer reading times in practically all measures that involved word integration processes, which reflect slow processing effects. This aspect is more noticeable at the integration level of low-frequency words, with a simultaneous word length interaction effect, since low-frequency medium and long-length words require integration times greater than those for low-frequency short-length words (L + LF_SPRT > M + LF_SPRT > S + LF_SPRT).

### Total Fixation Time (TFT)

**Table 9.** Median, 1^st^ and 3^rd^ percentiles, mean, standard deviation, and Kruskal-Wallis independent samples test: Total Fixation Time (in milliseconds) as a Function of Word Frequency and Word Length interaction in Dyslexia, Control and ADHD-I Groups.

IV	Groups	$\tilde{X}$ *(Q3–Q1)*	Mean (*SD*)	*K-S*
S + LF	Control	468.50 (738.00 – 316.75)	519.30 (354.66)	Control group ≠ ADHD-I^*^, Control group ≠ Dyslexia^*^
	DD	650.50 (1280.25 – 444.75)	935.55 (722.85)	
	ADHD-I	642.50 (906.00 – 410.25)	711.61 (526.91)	
S + MF	Control	344.50 (455.50 – 268.75)	416.49 (227.62)	Control group ≠ ADHD-I^*^, Control group ≠ Dyslexia^*^
	DD	536.00 (939.00 – 402.50)	737.53 (524.42)	
	ADHD-I	485.00 (778.50 – 318.75)	667.75 (559.83)	
M + LF	Control	684.50 (1122.50 – 468.00)	854.46 (615.81)	Control group ≠ ADHD-I^*^, Control group ≠ Dyslexia^*^
	DD	1266.00 (2148.75 – 676.00)	1608.22 (1372.13)	
	ADHD-I	881.50 (1626.00 – 505.75)	1274.68 (1057.19)	
M + MF	Control	588.50 (894.75 – 392.25)	689.37 (563.80)	Control group ≠ ADHD-I^*^, Control group ≠ Dyslexia^*^
	DD	1088.00 (1809.00 – 574.50)	1384.97 (1345.41)	
	ADHD-I	730.00 (1343.00 – 440.25)	991.35 (782.40)	
L + LF	Control	822.00 (1380.25 – 510.50)	1007.97 (788.13)	Control group ≠ ADHD-I^*^, Control group ≠ Dyslexia^*^
	DD	1553.50 (2873.25 – 788.00)	1885.74 (1508.13)	
	ADHD-I	1175.00 (1925.00 – 504.25)	1541.60 (1359.32)	
L + MF	Control	766.00 (1098.25 – 477.00)	853.32 (614.04)	Control group ≠ ADHD-I^*^, Control group ≠ Dyslexia^*^
	DD	1189.00 (2190.75 – 588.50)	1583.02 (1390.45)	
	ADHD-I	1003.00 (1732.00 – 542.75)	1414.46 (1173.34)	

Note. S = Short word. M = Medium word. L = Long word. LF = Low frequency. MF = Medium frequency. *K-W* = *Kruskal-Wallis* test. $\tilde{X}$ = Median. *Q3* = 3^rd^ percentile. *Q1*= 1^st^ percentile. *SD* = Standard deviation. Superscripts indicate significant group difference (*p* < 0.05); *p < 0 .005

Total Fixation Time (TFT) had a similar behaviour as SPRT, corroborating statistically significant differences between groups (See Table 9). Clearly, TFT is the variable that best distinguished all groups in all conditions: controls vs. children with dyslexia and controls vs. ADHD-I.

This finding is due to what this measure can reveal: visual word recognition cumulative processes, access to meaning, word integration in syntactic structures and in the mental representation of the text that is being constructed while reading. Subsequently, there is an interaction effect between word-frequency and word length (L + LF > M + LF > S + LF), with children with dyslexia having greater TFT’s, followed by children with ADHD-I and, finally, by the control group.

## Discussion

Firstly, it should be noted that all target words were expected to be fixated and that size and frequency should have an impact by increasing or decreasing the number of fixations, as well as increasing or decreasing the duration of fixations and regressions in each word. This performance will depend on the participants neuropsycholinguistic profile. The magnitude of this effect should vary according to the two lexical properties (less frequent / longer words, more fixations) studied, and be influenced by idiosyncratic group characteristics.

The eye movement dependent variables that best distinguished typical readers from children with dyslexia were: 1) Fixation Counts (FC); 2) First Pass Reading Times (FPRT) and 3) Second Pass Reading Times (SPRT).

Regarding the first variable, a global effect was found for all lexical properties studied, namely short and medium-frequency short words, low and medium-frequency medium words, and long-length low and medium-frequency words. Low-frequency short words activate more attentional mechanisms in children with dyslexia, since they are unknown or unfamiliar and, therefore require phonological decoding representations/mechanisms that may be of poor quality in this population.

In turn, the second variable, FPRT, highlights differences between medium-frequency short words, low and medium-frequency medium words and medium-frequency long words. SPRT had also a similar behaviour as FC and TFT, making it possible to find effects in all conditions evaluated.

In all the above-mentioned variables, children with dyslexia showed a poorer performance that was independent of their cognitive profile. Reading difficulties shown by dyslexic readers are better explained by deficits in phonological processing and by diminished activation speeds when accessing lexical pathway.

Regarding children with ADHD-I, the eye tracking variables that best distinguished them from typical readers were FC, FPRT, SPRT and, TFT. Regarding the first measure, compared to children with dyslexia, we found lexical properties effects only on medium-frequency short length words. As for the second measure, it was only possible to identify effects in one condition, namely long length medium-frequency words. For the third variable, effects were identified on four conditions, namely medium-frequency short and medium words and, low and medium-frequency long words. On the other hand, TFT emphasised effects in all lexical conditions studied, like what happens in children with dyslexia.

Regarding children with ADHD-I, only two ocular variables distinguished them from dyslexic readers, with the last group showing higher FC and longer SPRT. In the first measure, effects were found in three conditions, namely in medium and long low-frequency words and, in medium length medium-frequency words. As for the last, effects were identified only when the condition was short low-frequency words.

These findings allow us to conclude that eye movement differences between normative readers and children with ADHD-I are fewer than the ones found for typical readers compared to children with dyslexia, and that low-frequency long words were the ones that most affected both groups.

Furthermore, this investigation found very significant frequency-word and word-length effects between groups. Similar to the study done by Hyönä and Olson (36) and Tiffin-Richards and Schroeder (92), who found that the most difficult words to recognize, namely long length words and low-frequency words, received more fixations than relatively easier words to process, namely short length words and high frequency-words. Our research has also found that short and medium low-frequency words have an identical profile as long low-frequency words. The number of fixations they received were directly proportional to the word size, our data supports this finding unequivocally.

The effects of length and frequency were equally found both on the initial encounter with the word and on the frequency of regressions back to the target word. These effects were due to the lexical properties of more complex words which attracted multiple fixations on themselves. This effect, at the word-length level, was also observed in the studies mentioned earlier, as well as the frequency effect that also influences the duration of the initial fixation on the target word, which is higher for low-frequency words and lower for high-frequency words (36; 92).

The study by Hyönä and Olson (36) also found a very similar pattern of outcomes between dyslexic and typical readers. These authors concluded that, in both groups, fixation patterns during reading reflect momentary variations in relative ease of processing in a similar fashion to that observed in adults’ typical readers. These findings do not match our results which revealed statistically significant differences between groups according to the lexical properties of words, supporting the conclusions of other authors who found that children with dyslexia, compared to other readers, have qualitatively and quantitatively different eye movement patterns and ocular characteristics (19; 55; 107).

According to some authors (15; 19; 21, 22; 54), children with dyslexia exhibit erratic eye movements distributed almost randomly across the line of text, suggesting a deficiency in visual attentional processing and an immaturity of brain structures responsible for pursuit triggering. According to these authors, the largest quantitative and qualitative differences between children with dyslexia and typical readers lies in the size and number of regressions.

Our study confirms the findings of several authors, who concluded that children with dyslexia have higher number of regressions/revisits and, in turn, longer SPRT compared to typical readers, as well as higher number of fixations on target words (19; 31; 54). Our data also confirms Pavlidis's theory of oculomotor dysfunction (55), which predicts that children with dyslexia often make more fixations and more regressions/revisits in particular, along with shorter fixation durations. In other studies by the same author (52, 54), it has been shown that children with dyslexia make almost twice as many regressions as 6-year-old typical readers attending first grade. While 6-year-old typical readers performed regressive eye movements invariably smaller in size than the previous progressive saccade, the regressions of children with dyslexia usually appeared in groups of two or more and were often larger than the previous progressive saccade.

The finding in both Pavlidis's studies (52, 54) that high frequency of regressions, substantiated by their large size and erratic behaviour, makes them a decisive element in distinguishing children with dyslexia from other readers. Given the absence of differences between children with dyslexia and typical readers in their study, Hyönä and Olson (36) do not support the hypothesis of an oculomotor dysfunction. According to these authors, this data is consistent with the developmental delay theory.

Two decisions need to be made while reading with regard to readers' eye movements: how long to stay fixated at the present location and where to go next (36). Rayner and McConkie (69) found reasons to believe that these decisions are governed by independent mechanisms, namely automatic perceptual behaviours derived from experience, cognitive abilities, and lexical knowledge. Consequently, according to Hyönä and Olson (36), it could be argued that only one mechanism operates erroneously in children with dyslexia.

If the chunk involved in the duration works inappropriately, it means that the duration of the first fixation or the length of the gaze (i.e., the initial encounter with the word) will not reflect difficulties in word recognition. However, similarly to Hyönä and Olson (36) work, this notion was not supported by our data. Alternatively, it could be argued that the mechanism of reading redirection is not working properly. This change is involved in the visual deficit theory, which postulates a visual transient system defect sensitive to stimuli presented outside the foveal region (45).

Previous research has confirmed that extrafoveal information is used to determine where to go next in the text (18; 37; 70; 79). This type of deficit would be implicated in many regressive fixations and rereading’s in the absence of any processing difficulties. Contrary to Hyönä and Olson (36) study, our data on SPRT for correctly read words were not identical between children with dyslexia and typical readers. Moreover, the frequency of performing a regression immediately after an initial fixation on the target word was higher for children with dyslexia. Another finding corroborated by the same study was the presence of significant differences resulting from frequency-word effect on first and second pass reading times. In previous studies with adult readers, the effect appeared consistently at the level of first fixation durations.

The finding that word frequency affects the duration of the first fixation is consistent with the view that word frequency influences a relatively early phase of word processing (36). This effect was particularly observed in low-frequency words, which is consistent with the idea that only robust effects are reflected in first fixation durations. In previous studies, reinspection’s were not often analysed as a function of word frequency (36).

The few studies to investigate reinspection data (32; 88), have found significantly longer regressive fixation durations for low-frequency words than for high-frequency words, as in the present study. Furthermore, they found a similar but not significant trend in the number of fixations, unlike the present study which found a significant but equally identical trend.

The word-length effect was shown in the study by Hyönä and Olson (36) both by a larger number of fixations and, by longer FPRT and SPRT in long words, a finding also corroborated by our study. Like the study mentioned previously, first fixation duration was not influenced by word-length, which also happens in proficient reading (42; 69).

In the same study mentioned previously, long words attracted more fixations than medium and short words, a finding that was also supported by our study. At the time of the publication of the study by Hyönä and Olson (36), no word length influences on reinspection among typical readers had been observed. Carpenter and Daneman (16) also observed that word length did not correlate with the duration of regressive fixations. Our results point in the same direction as both studies mentioned above, as it can be attributed to the ability of typical readers to process words effortlessly, regardless of their size.

In the present study, we found an interaction between word length and frequency in all eye movement measurements used, namely FC, SFD, FPRT, SPRT and, TFT, which also corroborates the study of Hyönä and Olson (36). Hyönä and Olson (36) found a clearly significant word-frequency effect on FFD for short and long words, whereas in our study the effect was significant for medium and long words. According to these authors, there is no clear explanation for the absence of a frequency-word effect on medium-length words. The finding that medium-length low-frequency words tend to be slightly more frequent than other low-frequency words may, according to Hyönä and Olson (36), be a possible reason.

Our data identified an effect of frequency on medium-length words, supporting the explanation given by Hyönä and Olson (36). The finding that children with dyslexia have higher first fixation durations in medium-frequency long length words compared to low-frequency medium length words suggests that the latter were more familiar. However, the interaction was more easily interpretable considering first pass reading time. This variable reflects, in the study conducted by Hyönä and Olson (36), the finding that low-frequency long words capture a greater number of fixations on them.

Nevertheless, in the present study, the interaction between long and low frequency words relative to FPRT was not significant enough to discriminate the groups. In contrast, it was significant for medium-frequency short words, low and medium-frequency medium words, and medium-frequency long words. Among these, the highest FPRT were made by children with dyslexia at the low-frequency medium word level, followed by medium-frequency medium words.

Relatively to SPRT, low and medium-frequency words attracted considerably more reinspection’s than other words, corroborating once again Hyönä and Olson (36) work. These were among the least frequent words in the range of target words selected for this study.

Hyönä and Olson (36) stated that the presence of statistically significant differences at the FFD level makes this measure the one that presents the clearest and most general effects in terms of frequency and word length, but it was little discriminative in our study.

Our results show that FC, SPRT and TFT are the most comprehensive measures to study word frequency and word length effects; the last measure was not included in the study by Hyönä and Olson (36). According to Hyönä and Olson (36), SPRT was restricted to a subset of words. For them, the probability of going back to a word seems to be determined more by its frequency than by its length. However, in our study we found that children with dyslexia when dealing with low-frequency words have SPRT directly proportional to word size.

It is important to mention that the results obtained by Hyönä and Olson (36) were obtained through a reading aloud task, so they cannot be generalized to silent reading, as happens in our study. Unlike reading aloud, which implies a stronger link between eye movements occurring during reading and word recognition processes, silent reading is where reading differences between groups in oculomotor functioning is most likely to be observed. This data was later corroborated by Rayner (65) who found that the average fixation duration is shorter in silent reading, approximately 225 milliseconds compared to 275 milliseconds in reading aloud. Our study allows us to accept this possibility.

Regarding the effects of word length and word frequency in children with ADHD-I during silent reading, we did not find any study that addressed this theme. Children with ADHD-I are characterized by being unable to keep their attention on a continuous performance test, such as a task involving reading for understanding. This data is expressed by their high false alarm rates and increased reaction times (27). However, this inability to maintain attention is also shared with children with dyslexia (19). This striking feature was discovered by Pavlidis (55) in a group of children with dyslexia characterized by their inability to sustain fixation for more than a second on a task that involved following precisely and as fast as possible five light-emitting diodes (LEDs). It is important to note that the inability to accurately maintain fixation at a given point for one second or more has also been observed in other dyslexic studies (44; 98).

Finally, it can be concluded that reading performance in children with dyslexia is affected proportionally by the number of low-frequency words in the text, and the interaction between frequency and word size further contributes to a decreased reading fluency. This finding ultimately alters the degree of understanding given the allocation of a greater number of cognitive resources for processing the lexical complexity of words.

## Conclusion

The present study allowed us to conclude that children with dyslexia have cognitive profiles and eye movement patterns during reading that are qualitatively and quantitatively different from normative children and their peers with ADHD-I. The observation that children with ADHD-I also differ from dyslexic and typical readers in linguistic performance measures, whether in formal reading assessment or in eye tracking, points to the existence of different cognitive resources at the base of their reading problems.

This study supports the theory that in the genesis of developmental dyslexia there is a predominance of phonological impairments in comorbidity with other cognitive deficits, predominantly at the level of short-term verbal memory and verbal working memory. The use of eye tracking in this study allowed us the identification of interaction effects between word-length and word-frequency, characteristics that require identical cognitive resources, such as working memory, as well as, in some cases, oculomotor coordination, processing speed, short-term verbal memory, visual attention and ability to access lexical knowledge. The finding that children with dyslexia make, on average, more fixations, and regressions than typical readers support, in our opinion, the hypothesis that, in addition to being a phonological disorder, an oculomotor dysfunction and/or sequential incapacity coexist in dyslexia. This dysfunction also produces erratic eye movements and changes in visual perception of orderly and sequential text processing. However, while phonological processing is a necessary and ever-present condition for the decoding of any written word, regardless of its size or frequency, the oculomotor function is activated only during reinspection’s of words with certain lexical properties.

As for children with ADHD-I, we found that deficits in several cognitive functions, namely visual attention, lexical knowledge access, short-term verbal memory, short-term visual memory, visuospatial working memory, and processing speed were responsible for the neuropsycholinguistic difficulties manifested by these children. Unlike children with dyslexia, children with ADHD-I have a greater and more generalized number of cognitive shortfalls, which affect visual perception patterns and measures of linguistic performance. Our study supports the evidence that children with ADHD-I also have difficulties in phonological awareness, however, to a lesser degree than their dyslexic peers due to the absence of deficits at the level of verbal working memory. Another feature that allowed us to distinguish the reading profile of children with ADHD-I from those with dyslexia and to safely affirm that the reading problems of the former are triggered by different brain (dis)functions, was the complete absence of oculomotor dysfunctions in this group, regardless of the lexical property of the word. While oculomotor dysfunction seems to be a characteristic unique to dyslexia and observed only in words with certain lexical characteristics, the disturbance of visuospatial attention appears to be a specific property of ADHD-I, present in the decoding of any type of word.

The data gathered in this study confirms that in the origin of the different reading profiles observed in dyslexia and ADHD-I are multiple cognitive deficits, which supports the multiple cognitive deficits theory, which states that the reading difficulties encountered both in dyslexia and ADHD-I are explained by different cognitive underlying mechanisms. Within these deficits, there are those that are shared by both conditions, but of special importance for this study are the ones that would allow dyslexia to be distinguished from ADHD-I. To discover the specific neuropsycholinguistic limitations of each of these neurodevelopmental disorders, it was essential to record eye movements using eye tracking, which could be a tool with high diagnostic capacity in the future.

With the data gathered and presented in this work, in a future development of this study, we will show, using predictive modelling, that it is possible to move from group-level descriptions to individual-level predictions with high sensitivity and specificity, which is a first step towards making eye tracking a viable screening method.

Finally, it is important to highlight some limitations of the present study, namely the sample size and the problems related to the differential diagnosis and give indications of future directions for this research. In relation to the first, in a future edition of this work, we intend to increase sample size to reinforce the generalization of our conclusions to the studied target populations. As for the second limitation, we were not immune to the limitations encountered in other studies when selecting participants and assign them by clinical groups, since there is a high comorbidity between both disorders. We believe that many of the doubts and wrong conclusions that have arisen in other investigations regarding the sharing of the same cognitive deficits by both clinical conditions are due to misdiagnosis at the stage of selecting participants. In our study, we believe that the identification of neuropsycholinguistic traits distinct from children with dyslexia and children with ADHD-I helped to mitigate the effects of comorbidity.

### Ethics and Conflict of Interest

The author(s) declare(s) that the contents of the article are in agreement with the ethics described in http://biblio.unibe.ch/portale/elibrary/BOP/jemr/ethics.html and that there is no conflict of interest regarding the publication of this paper.

### Acknowledgements

This work has been funded by the Fundação para a Ciência e a Tecnologia (FCT, Foundation for Science and Technology), grant UIDB/00214/2020.

## Appendix

**Table 10.** Means, standard deviations, median, 1^st^ and 3^rd^ percentiles, ANOVA and *Kruskal-Wallis* independent samples: WISC-III composite results.

Measures	Groups	Mean (*SD*)	$\tilde{X}$(*Q3-Q1*)	Multiple Comparisons
Verbal IQ	Control	102.85 (13.20)		Control ≠ ADHD-I (*p*=0.000)^1.2^ , Dyslexia ≠ ADHD-I (*p*=.002)^1.2^
	Dyslexia	98.89 (13.14)		
	ADHD-I	83.45 (13.02)		
Performance IQ	Control		106.00 (115.50-87.00)	ADHD-I ≠ Dyslexia (*p*=0.000)^5^ , ADHD-I ≠ Control (*p*=0.000)^5^
	Dyslexia		95.00 (105.00-90.00)	
	ADHD-I		79.50 (89.00-73.00)	
Full IQ	Control		98.00 (117.00-89.50)	ADHD-I ≠ Dyslexia (*p*=0.000)^5^ , ADHD-I ≠ Control (*p*=0.000)^5^
	Dyslexia		98.00 (105.00-88.00)	
	ADHD-I		78.00 (84.75-74.25)	
Verbal Comprehension Index (VCI)	Control	104.08 (13.43)		Control ≠ ADHD-I (*p*=0.000)^1.2^ , Dyslexia ≠ ADHD-I (*p*=0.001)^1.2^
	Dyslexia	100.58 (11.72)		
	ADHD-I	84.50 (13.93)		
Perceptual Organization Index (VSI)	Control		106.00 (118.00-91.50)	ADHD-I ≠ Dyslexia (*p*=0.009)^5^ , ADHD-I ≠ Control (*p*=0.001)^5^
	Dyslexia		98.00 (103.00-86.00)	
	ADHD-I		87.00 (91.00-72.00)	
Processing Speed Index (PSI)	Control	102.31 (21.67)		Dyslexia ≠ ADHD-I (*p*=0.002)^3.4^
	Dyslexia	102.68 (13.78)		
	ADHD-I	87.37 (10.89)		

Note: ^1^*ANOVA F* Test; ^2^*Tukey HSD*; ^3^*Brown-Forsythe* statistic; ^4^*Games-Howell*;^5^*Kruskal-Wallis* independent samples; *SD* – Standard deviation; $\tilde{X}$ – Median, *Q3* – 3^rd^ percentile, *Q1* – 1^st^ percentile.

**Table 11.** Means, standard deviations, median, 1^st^ and 3^rd^ percentiles, ANOVA and *Kruskal-Wallis* independent samples: WISC-III subtest results.

Indexes	Subtests	Groups	Mean (*SD*)	$\tilde{X}$(*Q3-Q1*)	Multiple Comparisons
Verbal Comprehension	Vocabulary	Control		10.00 (14.00-9.00)	ADHD-I ≠ Dyslexia (*p*=0.000)^5^ , ADHD-I ≠ Control (*p*=0.000)^5^
		Dyslexia		10.00 (12.00-9.00)	
		ADHD-I		6.00 (8.00-5.00)	
	Information	Control		9.00 (11.00-7.00)	ADHD-I ≠ Control (*p*=0.036)^5^
		Dyslexia		8.00 (11.00-6.00)	
		ADHD-I		6.00 (9.00-6.00)	
	Similarities	Control		13.00 (14.00-9.50)	ADHD-I ≠ Dyslexia (*p*=0.036)^5^
		Dyslexia		12.00 (13.00-11.00)	
		ADHD-I		9.00 (11.50-8.00)	
	Comprehension	Control	10.46 (2.47)		n.s.
		Dyslexia	9.94 (1.95)		
		ADHD-I	7.39 (2.45)		
Perceptual Organization	Block Design	Control	10.92 (2.53)		Control ≠ ADHD-I (*p*=0.000)^1.2^ , Dyslexia ≠ADHD-I (*p*=0.000)^1.2^
		Dyslexia	9.59 (2.15)		
		ADHD-I	6.44 (2.75)		
	Picture Arrangement	Control		9.00 (11.50-7.50)	ADHD-I ≠ Control (*p*=0.041)^5^ , ADHD-I ≠ Dyslexia (*p*=0.006)^5^
		Dyslexia		9.00 (10.00-8.00)	
		ADHD-I		7.50 (9.00-4.25)	
	Picture Completion	Control	11.08 (3.20)		Control ≠ ADHD-I (*p*=0.012) ^1.2^
		Dyslexia	10.24 (2.73)		
		ADHD-I	8.56 (2.41)		
	Object Assembly	Control	9.92 (3.30)		n.s.
		Dyslexia	10.47 (3.20)		
		ADHD-I	8.72 (2.08)		
Processing Speed	Coding	Control	11.08 (3.88)		Control ≠ ADHD-I (*p*=0.019)^3.4^ , Dyslexia ≠ ADHD-I (*p*=0.015)^3.4^
		Dyslexia	9.82 (2.63)		
		ADHD-I	7.39 (2.25)		
	Symbol Search	Control	9.77 (4.38)		Dyslexia ≠ ADHD-I (*p*=0.003)^3.4^
		Dyslexia	11.53 (2.94)		
		ADHD-I	7.89 (2.14)		
Working Memory	Digit Span Total	Control	10.62 (2.43)		Control ≠ Dyslexia (*p*=0.023)^1.2^ , Control ≠ ADHD-I (*p*=0.027)^1.2^
		Dyslexia	8.42 (2.06)		
		ADHD-I	7.40 (2.26)		
	Arithmetic	Control	9.85 (3.11)		n.s.
		Dyslexia	9.18 (2.83)		
		ADHD-I	8.17 (1.98)		
	Mazes	Control	11.31 (2.98)		n.s.
		Dyslexia	11.56 (2.28)		
		ADHD-I	9.68 (3.16)		

Note. n.s. – not statistically significant. ^1^*ANOVA F* test; ^2^*Tukey HSD*; ^3^*Brown-Forsythe* Statistic; ^4^*Games-Howell*;^5^*Kruskal-Wallis* independent samples. *SD* – Standard deviation; $\tilde{X}$ – Median, *Q3* – 3^rd^ percentile, *Q1* – 1^st^ percentile.

**Table 12.** Means, standard deviations, median, 1^st^ and 3^rd^ percentiles, ANOVA and *Kruskal-Wallis* independent samples: Coloured Progressive Matrices (CPM).

Items	Groups	Mean (SD)	$\tilde{X}$(*Q3–Q1)*	Multiple Comparisons
Total	Control	30.44 (2.78)		Control ≠ ADHD-I (*p*=0.009)^3^
	Dyslexia	28.47 (4.15)		
	ADHD-I	26.43 (4.87)		
Set A	Control	10.06 (1.03)		n.s.
	Dyslexia	9.84 (0.96)		
	ADHD-I	9.43 (1.60)		
Set B	Control		11.00 (12.00-10.00)	ADHD-I ≠ Control (*p*=0.005)^5^
	Dyslexia		9.00 (11.00-8.00)	
	ADHD-I		9.00 (10.00-6.50)	
Set AB	Control	9.53 (2.87)		n.s.
	Dyslexia	9.58 (2.12)		
	ADHD-I	8.57 (2.58)		

Note: n.s. – not statistically significant. ^3^*Brown-Forsythe* statistic; ^5^*Kruskal-Wallis* independent samples; *SD* – Standard deviation; ^7^ $\tilde{X}$ – Median, *Q3* – 3^rd^ percentile, *Q1* – 1^st^ percentile.

**Table 13.** Means and standard deviations: Rey Complex Figure Test.

Items	Groups	Mean (SD)	Multiple Comparisons
Copy score	Control	25.50 (5.68)	n.s.
	Dyslexia	26.08 (6.52)	
	ADHD-I	21.50 (7.11)	
Copy (time of execution in seconds)	Control	293.33 (78.75)	
	Dyslexia	318.28 (111.72)	
	ADHD-I	341.44 (119.45)	
Memory score	Control	10.13 (6.14)	
	Dyslexia	11.97 (4.24)	
	ADHD-I	8.55 (5.46)	
Memory (time of execution in seconds)	Control	158.45 (87.48)	
	Dyslexia	210.00 (109.79)	
	ADHD-I	150.22 (55.80)	

Note: n.s. – not statistically significant.

**Table 14.** 1^st^ and 3^rd^ percentiles and *Kruskal-Wallis* independent samples: O Rei – Reading Fluency and Accuracy Test

Variables	Groups	$\tilde{X}$(*Q3–Q1)*	Multiple Comparisons
Number of correct words read in 1 minute	Control	111.00 (125.00-99.50)	Dyslexia ≠ Control (*p*=0.000)^5^ , ADHD-I ≠ Control (*p*=0.000)^5^
	Dyslexia	71.00 (80.00-50.50)	
	ADHD-I	77.00 (85.00-46.50)	
Number of correct words read in 3 minutes	Control	270.00 (274.50-265.50)	Dyslexia ≠ Control (*p*=0.000)^5^ , ADHD-I ≠ Control (*p*=0.001)^5^
	Dyslexia	179.00 (227.50-123.50)	
	ADHD-I	177.00 (242.50-111.50)	
Total reading time (in seconds)	Control	153.00 (176.50-133.00)	ADHD-I ≠ Control (*p*=0.001)^5^ , Dyslexia ≠ Control (*p*=0.000)^5^
	Dyslexia	256.00 (381.50-211.00)	
	ADHD-I	235.00 (399.50-186.50)	
Fluency Index	Control	90.00 (91.50-88.50)	Dyslexia ≠ Control (*p*=0.000)^5^ , ADHD-I ≠ Control (*p*=0.000)^5^
	Dyslexia	59.67 (75.83-41.17)	
	ADHD-I	59.00 (80.83-37.17)	

Note. ^5^*Kruskal-Wallis* independent samples; $\tilde{X}$ – Median, *Q3* – 3^rd^ percentile, *Q1* – 1^st^ percentile.

**Table 15.** Means, standard deviations and ANOVA independent samples test: TCL – Reading Comprehension Test.

Variables	Groups	Mean (*SD*)	Multiple Comparisons
Literal Comprehension	Control	6.93 (2.09)	Control ≠ Dyslexia (*p*=0.026)^1.2^
	Dyslexia	4.73 (2.12)	
	ADHD-I	5.50 (2.31)	
Inferential Comprehension	Control	4.71 (1.77)	n.s.
	Dyslexia	4.33 (2.29)	
	ADHD-I	3.38 (1.54)	
Critical Comprehension	Control	1.07 (0.62)	
	Dyslexia	1.27 (0.70)	
	ADHD-I	0.81 (0.75)	
Reorganization	Control	2.71 (0.99)	
	Dyslexia	1.60 (1.30)	
	ADHD-I	2.13 (1.20)	
Total Score	Control	16.07 (5.08)	Control ≠ ADHD-I (*p*=0.033)^1.2^
	Dyslexia	11.93 (4.85)	
	ADHD-I	11.50 (4.75)	

Note. n.s. – not statistically significant. ^1^ *ANOVA F* test; ^2^*Tukey HSD*; *SD* – Standard Deviation.
